# Comparison of *n*-butyl-2-cyanoacrylate and polyvinyl alcohol particles for bronchial artery embolisation in primary lung cancer: a retrospective cohort study

**DOI:** 10.1186/s12931-022-02183-7

**Published:** 2022-09-20

**Authors:** Jae Hwan Lee, Chang Jin Yoon, Yun Su Jung, Won Seok Choi, Chong-ho Lee, Guy Mok Lee

**Affiliations:** 1 Department of Radiology, Seoul National University College of Medicine, Seoul National University Bundang Hospital, 82, Gumi-ro 173beon-gil, Bundang-gu, Seongnam-si, Gyeonggi-do 13620 South Korea; 2 Seoul National University Bundang Hospital, 82, Gumi-ro 173beon-gil, Bundang-gu, Seongnam-si, Gyeonggi-do 13620 South Korea; 3 Seoul National University Hospital, 101, Daehak-ro, Jongno-gu, Seoul, 03080 South Korea

**Keywords:** Bronchial artery embolisation, Haemoptysis, *n*-butyl-2-cyanoacrylate, Primary lung cancer

## Abstract

**Background:**

Bronchial artery embolisation (BAE) is an effective treatment option to control haemoptysis in primary lung cancer. However, no studies have investigated optimal embolisation material for BAE in lung cancer patients. Thus, this study aimed to compare the safety and efficacy of BAE performed using *n*-butyl-2-cyanoacrylate (NBCA) and polyvinyl alcohol (PVA) particles in primary lung cancer patients to determine which embolic material is better for patients with haemoptysis.

**Methods:**

This retrospective study was approved by the institutional review board, and consent was waived. The rates of hemostasis, complications, procedure time, dose–area product, and haemoptysis-free survival were retrospectively compared between primary lung cancer (non-small cell [n = 111] and small cell [n = 11]) patients who underwent BAE using NBCA (n = 58) or PVA particles (n = 64) between January 2004 and December 2019. Predictors of recurrent haemoptysis were analysed using the Cox proportional hazard regression model.

**Results:**

Among 122 patients (mean age, 66 ± 10 years; range 32–86 years; 103 men), more patients in the NBCA group (81.0%; 47 of 58) achieved complete hemostasis than did patients in the PVA group (53.1%; 34 of 64) (*P* = 0.002). No major complications were observed in either group. The procedure time (36.4 ± 21.6 vs. 56.3 ± 27.4 min, *P* < 0.001) was shorter, and the dose–area product (58.6 ± 64.0 vs. 233.5 ± 225.0 Gy*cm^2^, *P* < 0.001) was smaller in the NBCA group than in the PVA group. The median haemoptysis-free survival was 173.0 in the NBCA group compared with 20.0 days in the PVA group (*P* < 0.001). The PVA use (*P* < 0.001) and coagulopathy (*P* = 0.014) were independent predictors of shortened haemoptysis-free survival.

**Conclusion:**

BAE using NBCA showed significantly superior initial hemostasis with longer haemoptysis-free survival, shorter procedure time, and reduced radiation dose than BAE using PVA particles. The PVA use and coagulopathy were independent predictors of recurrent haemoptysis.

*Trial registration*: Retrospectively registered

## Background

Bronchial artery embolisation (BAE) is a safe and effective treatment for haemoptysis [1–9]. Most previous investigations have focused on the usefulness of BAE for benign diseases, such as bronchiectasis, tuberculosis, and aspergillosis [1–3, 7, 8]. Based on the literature, up to 30% of primary lung cancer patients develop haemoptysis, with 10% experiencing massive haemoptysis [4, 5]. A few studies on BAE for haemoptysis due to malignant disease have reported a relatively low clinical success rate compared with those with benign disease [4–6, 10, 11]. However, these are limited by a small study population [4], heterogeneous disease entity encompassing primary to secondary malignancy [5, 10–12], and lack of standardised use of embolic materials [4, 5, 10–12].

Particulate embolic agents such as polyvinyl alcohol (PVA), absorbable gelatin sponge particles, and tris-acyl microspheres have been widely used for BAE. Clinical outcomes among patients undergoing embolisation with different particles are similar [2, 13]; however, there is no evidence or consensus regarding the embolic material [1, 2, 7].

The interest in BAE using *n*-butyl-2-cyanoacrylate (NBCA) has recently increased, owing to several advantages offered by this agent; these include rapid and permanent vessel embolisation, controllable polymerisation rates (by adjusting the ratio of NBCA to iodised oil), and relatively short procedure time [1, 3, 7, 9]. Yoo et al. [1] showed that the use of NBCA in BAE was feasible and effective in 108 patients with benign diseases. Woo et al. [7] also showed that NBCA was a better embolic material than PVA particles in preventing recurrent haemoptysis. However, to the best of our knowledge, no studies have focused on BAE performed using NBCA for controlling haemoptysis in patients with primary lung cancer. Therefore, this study aimed to retrospectively compare the safety and efficacy of BAE using NBCA and PVA particles in patients with primary lung cancer patients and to determine which embolic material (NBCA or PVA particles) is better for patients with haemoptysis.

## Materials and methods

### Patients

The institutional review board of the Seoul National University Bundang Hospital approved this retrospective study (B-2008-631-111). The requirement for informed consent was waived. The baseline characteristics of both groups are shown in Table 1. This study included patients with primary lung cancer who underwent BAE between March 2004 and December 2019. Primary lung cancer was diagnosed via percutaneous or bronchoscopic biopsy, and staging was determined using chest computed tomography (CT), position emission tomography, and bone scan. The exclusion criteria were as follows: previous history of BAE before the diagnosis of lung cancer; BAE after curative treatment of lung cancer with no residual disease; other concomitant cancers, non-compliance with follow-up within 1 month after BAE; unclear cause of haemoptysis owing to concomitant bronchiectasis, tuberculosis, or aspergilloma; missing data from the electronic medical records; and post-biopsy bleeding. A computerised keyword-based search of electronic medical records was conducted to obtain data related to demographics, cancer, and haemoptysis of the NBCA and PVA groups; patients were selected consecutively.

**Table 1:** Baseline characteristics of patients with primary lung cancer who underwent BAE using NBCA and PVA particles

Parameter	NBCA group (n = 58)	PVA group (n = 64)	*P*-value
Age^*^ (y)	66.5 ± 10.9	65.8 ± 10.3	0.532
Male	47 (81.0)	56 (87.5)	0.454
Haemoptysis grade			0.112
Grade 3 (> 300 mL)	11 (19.0)	23 (35.9)	
Grade 2 (100–300 mL)	35 (60.3)	31 (48.4)	
Grade 1 (≤ 100 mL)	12 (20.7)	10 (15.6)	
Cancer histology (n, %)			0.294
Adenocarcinoma	20 (34.5)	22 (34.4)	
Squamous cell carcinoma	23 (39.7)	27 (42.2)	
“Not otherwise specified” non-small cell lung cancer	7 (12.1)	12 (18.8)	
Small cell lung cancer	8 (13.8)	3 (4.7)	
Stage			0.848
IA	2 (3.4)	1 (1.6)	
IB	2 (3.4)	5 (7.8)	
IIA	2 (3.4)	2 (3.1)	
IIB	3 (5.2)	2 (3.1)	
IIIA	7 (12.1)	10 (15.7)	
IIIB	14 (24.1)	13 (20.3)	
IV	28 (48.3)	31 (48.4)	
Mean maximal tumour diameter (mm)^*^	57.8 ± 30.3	55.6 ± 23.8	0.650
Mass with cavitary lesion	11 (19.0)	8 (12.5)	0.325
Tumour location			0.313
Central	39 (67.2)	49 (76.6)	
Peripheral	19 (32.8)	15 (23.4)	
Coagulopathy	6 (10.3)	3 (4.8)	0.309
Hemodynamic instability	10 (17.2)	5 (7.8)	0.167
Previous anticancer treatment			0.492
Chemotherapy only	21 (36.2)	28 (43.8)	
Chemoradiation	7 (12.1)	4 (6.2)	
Radiation only	14 (24.1)	17 (26.6)	
Surgery	5 (8.6)	8 (12.5)	
No treatment	11 (19.0)	7 (10.9)	

Data indicate number of patients; percentages are in parenthesis

BAE: bronchial artery embolisation; NBCA: *n*-butyl-2-cyanoacrylate; PVA: polyvinyl alcohol

^*^Data are presented as means ± standard deviation

### BAE procedures

All BAE procedures were performed by one of four interventional radiologists (J.H.L., C.J.Y., H.C.K., and H.J.J.). The type of embolic agents (PVA or NBCA) was randomly selected at the discretion of the attending interventional radiologists. Both embolic agents were routinely used during the study without a pause or transition period. After a right or left femoral arterial access was obtained with a 5-F vascular sheath, thoracic aortography was performed using a 5-F pigtail catheter (Royal Flush®; Cook Medical, Bloomington, IN, USA) with the tip placed at the ascending aorta. Selective angiography of the bronchial and non-bronchial systemic collateral arteries was performed using shaped 5-F angiographic catheters (GRB, Cobra, Headhunter; Cook Medical). The following angiographic findings were considered pathologic: tumour blush, tortuous tumour-feeding arteries, and bronchopulmonary shunting [6, 7]. All pathologic bronchial and non-bronchial systemic arteries were embolised. A 2- or 3-F microcatheter (Progreat, Terumo, Tokyo, Japan; Microferret, Cook Medical; Renegade, Boston Scientific, Natick, MA, USA) was co-axially introduced and advanced as distally as possible to avoid reflux of embolic agents into the spinal arteries or the aorta.

**Fig. 1:**
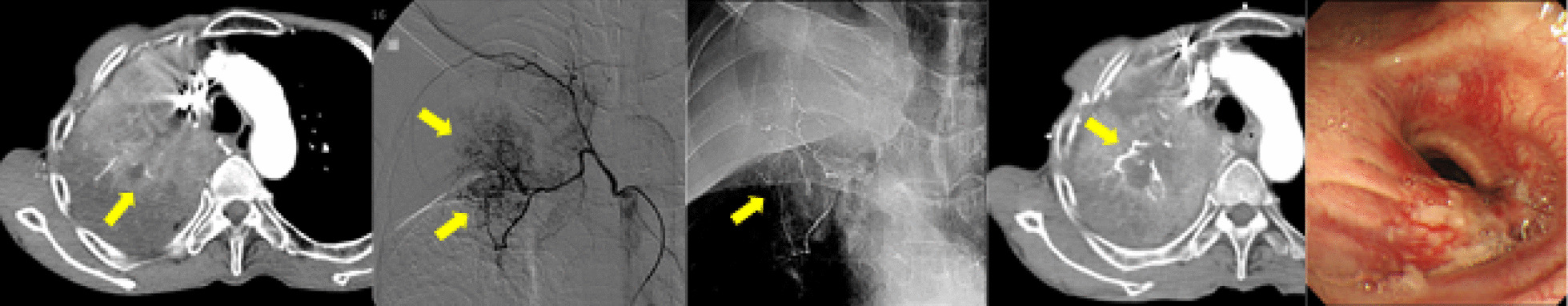
Images from a 72-year-old man with right lung adenocarcinoma having moderate haemoptysis. **a** Axial enhanced CT scan before bronchial artery embolisation shows a large mass in the right upper lung (arrow). **b** Angiography of the right intercostobronchial trunk shows multiple small feeding arteries with fine tumour staining (arrows). **c** After embolisation using a glue-lipiodol mixture, spot radiography shows NBCA/lipiodol casting of the tumour-feeding arteries (arrow). **d** 1-month follow-up chest CT scan shows residual NBCA/lipiodol densities in the tumour (arrow). **e** On 1-month follow-up bronchoscopy, hyperemic right main lobar bronchus was noted due to tumour infiltration, without evidence of necrosis. NBCA: *n*-butyl-2-cyanoacrylate; PVA: polyvinyl alcohol

In the PVA group, 355–500 μm PVA particles (Contour PVA Embolisation particles, Boston Scientific; PVA foam embolisation particles, Cook Medical) were diluted with 5 mL of saline and 15 mL of contrast in a 20-mL syringe connected to a 1-mL delivery syringe via a three-way stopcock [2, 6, 7]. The endpoint of embolisation was a complete cessation of the forward flow of the opacified embolic solution. In the NBCA group, NBCA was diluted with iodised oil (Lipiodol Ultra Fluide, Guerbet, Roissy, France) at a ratio of 1:3 to 1:5. After flushing the microcatheter with 5% dextrose solution, 0.5–2 mL of the mixture was carefully administered under fluoroscopy [7]. The mixing ratio, injection volume, and rate were determined based on the target vessel size and blood flow. After injection of the mixture, the microcatheter was quickly removed to prevent adhesions of the catheter tip to the vessel. A new microcatheter was prepared for each embolisation using NBCA if there was more than one culprit vessel. After embolisation with PVA or NBCA, ascending thoracic aortography was performed to confirm the disappearance of all culprit vessels (Fig. 1). The procedure time and dose–area product for each procedure were recorded [14].

### Follow-up

After BAE, all patients were monitored for at least 2 days in the intensive care unit or ward. If haemoptysis persisted or recurred during hospitalisation, bronchoscopy and/or repeated BAE were performed at the attending physician’s discretion. After the haemoptysis resolved for at least 2 days, the patient was discharged. After discharge, the patient visited the outpatient clinic within 1 month of the onset of haemoptysis. Regular follow-up for lung cancer management was conducted every 1–2 months. Follow-up laboratory tests and chest CT were performed at 2–4-month intervals, and anticancer treatment was administered as appropriate. If haemoptysis recurred, the patient visited the emergency department. A multidisciplinary team comprising emergency physicians, pulmonologists, hemato-oncologists, and interventional radiologists determined the optimal treatment, including medication, bronchoscopy, or repeated treatment BAE.

### Definition and analysis

The comparison of technical and clinical outcomes (rates of haemostasis, haemoptysis-free survival, and complication) between the two groups was the main interest of this study. Technical success was defined as complete embolisation of the bronchial and non-bronchial collateral vessels in which embolisation was attempted [7]. Based on previous reports [7, 18], haemoptysis was graded as a single episode of massive bleeding (> 300 mL/d; grade 3), moderate bleeding (> 100 mL/d; grade 2), or a small amount of bleeding ($\le$ 100 mL/d; grade 1). Clinical outcome was categorised as success (complete cessation of haemoptysis within 24 h of BAE and no recurrence until discharge) or failure (persistent or recurrent haemoptysis during the admission period) [6]. Haemoptysis-free survival (HFS) was defined as the time from BAE until recurrent haemoptysis or death. To evaluate complications, follow-up CT and/or bronchoscopy findings after BAE were reviewed to assess pulmonary ischaemia, infarction, or bronchial abnormalities. Grade I–V adverse events were determined using the Common Terminology Criteria for Adverse Events version 5.0. All patients underwent enhanced CT 1 or 2 days before BAE. Tumour size and location were also identified. Tumour size was determined by the maximal diameter in the axial image. Tumour location was dichotomised into central and peripheral positions. The central location was defined as the primary tumour abutting the mediastinum, and the other was defined as the peripheral position [6]. Two authors (J.H.L and C.J.Y.), blinded to the embolic agent and clinical outcomes, interpreted the CT images. Coagulopathy was defined as prothrombin time exceeding 1.5-fold the upper limit of normal or platelet counts < 50 × 10^9^/L [15, 16]. Hemodynamic instability was defined as systolic blood pressure < 80 mmHg, diastolic blood pressure < 50 mmHg, or heart rate < 50 beats per minute [17].

### Statistical analysis

Data are presented as means and standard deviations for parametric variables and absolute numbers and percentages for nonparametric variables. The baseline characteristics of both groups were compared using Student’s *t*-test for continuous variables and χ^2^ analysis for categorical variables. Technical and clinical success, and overall and major complication rates, were evaluated using Fisher’s exact test. The HFS rates were analysed using Kaplan–Meier estimates and compared using the log-rank test. Prognostic factors for recurrent haemoptysis were evaluated using a Cox proportional hazards model. Potential predictors (*P* < 0.1) of HFS were included in the multivariable analysis. Outcomes are shown as hazard ratios and 95% confidence intervals. *P*-values less than 0.05 were considered significant. Statistical analysis was performed using MedCalc (version 14.0) and IBM PASW statistics software for Windows (version 18.0; SPSS, Chicago, IL, USA).

## Results

### Patients

**Fig. 2:**
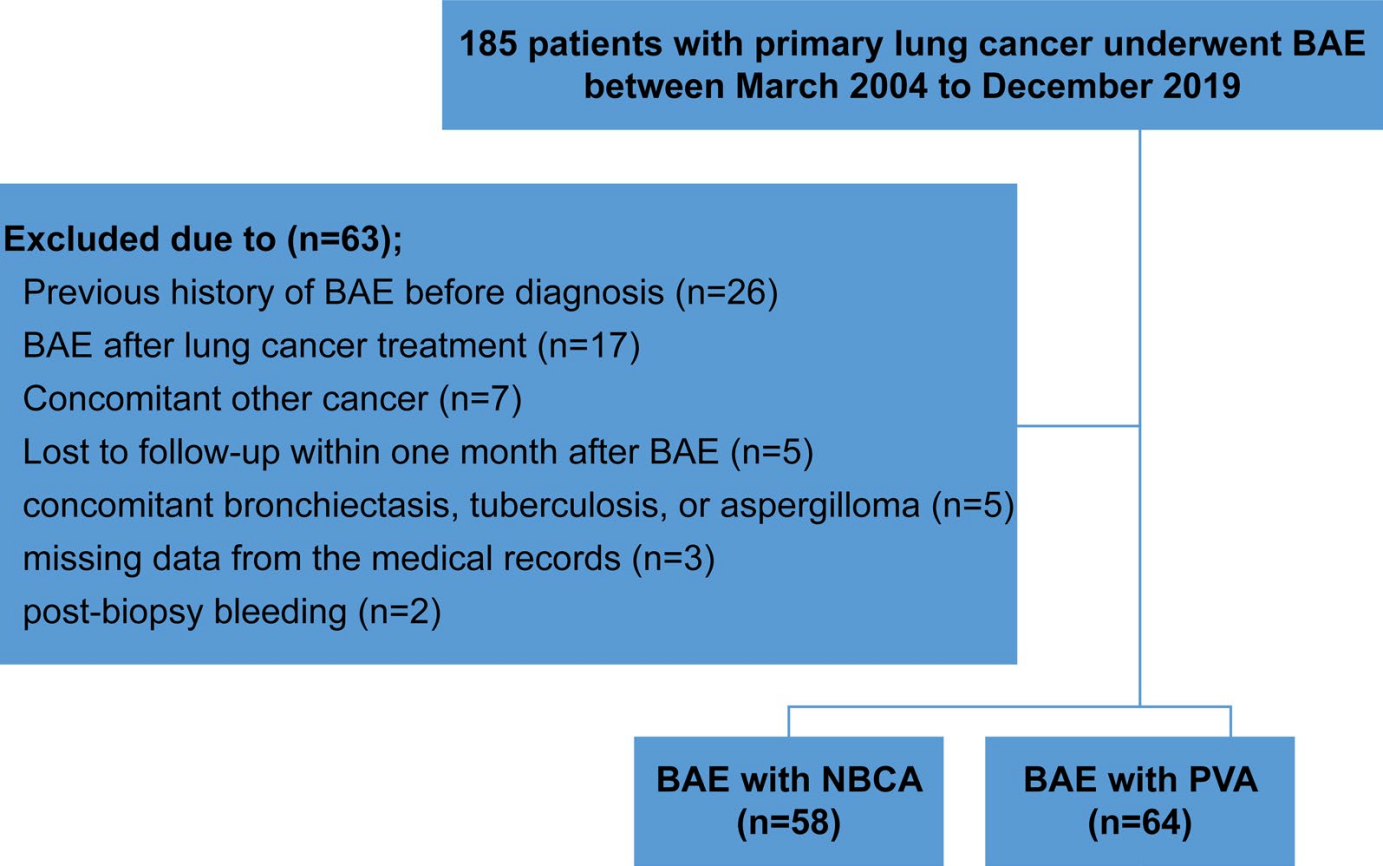
Flowchart showing patient selection. BAE: bronchial artery embolisation; NBCA: *n*-butyl-2-cyanoacrylate; PVA: polyvinyl alcohol

Overall, 122 patients (mean age, 66 ± 10; range 32–86 years) comprising 103 men (mean age, 65 ± 10; range 32–86 years) and 19 women (mean age, 69 ± 8; range 52–82 years) who underwent BAE with NBCA (n = 58) or PVA particles (n = 64) were included (Fig. 2). Significant differences were not observed between the groups in terms of age, sex, amount of haemoptysis, stage and histologic type of cancer, tumour size, and location. Reasons for exclusion were a previous history of BAE before being diagnosed with lung cancer (n = 26); BAE after curative treatment of lung cancer with no residual disease (n = 17); other concomitant cancers (n = 7); non-compliance with follow-up within 1 month after BAE (n = 5); unclear cause of haemoptysis owing to concomitant bronchiectasis, tuberculosis, or aspergilloma (n = 5); missing data from the electronic medical records (n = 3); and post-biopsy bleeding (n = 2).

Table 2 shows the angiographic findings and outcomes of BAE in both groups. Tumour blush was noted in all patients in both groups. No significant difference was found in angiographic findings between the NBCA and PVA groups. In total, 246 arteries (per-patient average of 2.02 ± 0.93 arteries) were embolised. No significant difference was noted in the number of embolised vessels between the two groups (*P* = 0.169).

**Table 2:** Angiographic findings and outcomes of BAE

Finding	NBCA group (n = 58)	PVA group (n = 64)	*P*-value
Angiographic findings			
Tumour blush	58 (100)	64 (100)	0.999
Tortuous arteries	14 (24.1)	14 (21.9)	0.831
Bronchopulmonary shunt	10 (17.2)	9 (14.1)	0.815
Number of embolised vessels ^*^	2.1 ± 1.0	1.9 ± 0.8	0.169
Technical success	57 (98.3)	61 (95.3)	0.621
Clinical success	47 (81.0)	34 (53.1)	0.002
Procedure time (min)^*^	36.4 ± 21.6	56.3 ± 27.4	< 0.001
Dose–area product (Gy * cm^2^)	58.6 ± 64.0	233.5 ± 225.0	< 0.001

Data indicate number of patients; percentages are in parentheses

BAE: bronchial artery embolisation; NBCA: *n*-butyl-2-cyanoacrylate; PVA: polyvinyl alcohol

^*^Data are presented as means ± standard deviation

### Technical and clinical outcomes

BAE was technically successful in 98.3% (57 of 58) and 95.3% (61 of 64) of the patients in the NBCA and PVA groups, respectively (*P* = 0.621). Technical failures occurred because of the tortuosity of the culprit artery (NBCA group, n = 1; PVA group, n = 2) and stenosis of the orifice (PVA group, n = 1). On analysing clinical outcomes, the number of patients who achieved successful hemostasis was higher in the NBCA group than in the PVA group (47 of 58; 81.0% vs. 34 of 64; 53.1%; *P* = 0.002). Among patients with failed hemostasis after the initial BAE, six patients (NBCA group, n = 2; PVA group, n = 4) underwent repeat BAE (n = 5) or bronchoscopy (n = 1) but died due to persistent haemoptysis. Among the nine patients with coagulopathy before BAE, clinical success was achieved in 66.6% (4 of 6) in the NBCA group and 33.3% (1 of 3) in the PVA group (*P* = 0.610).

The procedure time was shorter in the NBCA group than in the PVA group (36.4 ± 21.6 vs. 56.3 ± 27.4 min; *P* < 0.001). The dose–area product was 58.6 ± 64.0 Gy * cm^2^ in the NBCA group compared with 233.5 ± 225.0 Gy * cm^2^ in the PVA group (*P* < 0.001).

No adverse events of ≥ grade 3, as per the Common Terminology Criteria for Adverse Events, were noted in both groups. Three patients in the NBCA group and two patients in the PVA group complained of chest pain after BAE, which was resolved by intravenous or oral administration of analgesics. On follow-up, CT (n = 110) and/or bronchoscopy (n = 46) were performed within 3 months after BAE. No procedure-related complications, such as pulmonary or bronchial infarction, were observed in either group.

### Recurrent haemoptysis, survival, and predictor

**Fig. 3:**
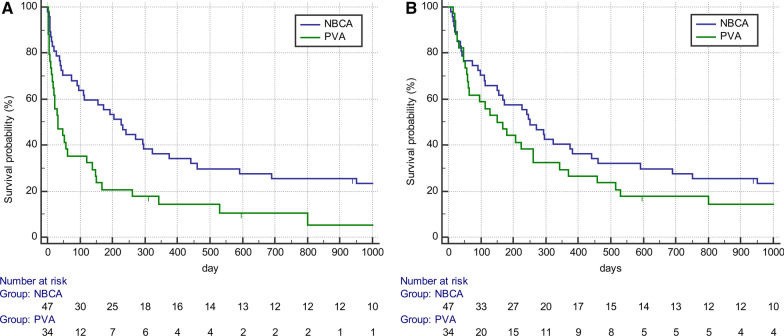
**a** Kaplan–Meier curves of haemoptysis-free survival in 81 patients with clinically successful haemoptysis after bronchial artery embolisation, stratified based on the type of embolic material. **b** Kaplan–Meier curves of overall survival in 81 patients with clinically successful haemoptysis after bronchial artery embolisation, stratified based on the type of embolic material

The mean follow-up durations for the NBCA and PVA groups were 594.9 (range 4–4,603; median, 202.0) days and 98.4 (range 4–2,218; median, 100.0) days, respectively. Haemoptysis recurred in 42 patients (9 of 56 vs. 33 of 60 in the NBCA vs. PVA groups; *P* = 0.004), which was managed by medication (n = 33), repeated BAE (n = 7), and bronchoscopy (n = 2). Three (33.3%) patients in the NBCA group and 27 (81.8%) patients in the PVA group experienced recurrent haemoptysis within 30 days after BAE. The HFS rates of the NBCA group were significantly higher than those of the PVA group (median survival 176.0 vs. 16.5 days; *P* < 0.001) (Fig. 3a).

During follow-up, 106 patients died (39 vs. 57 in the NBCA vs. PVA groups; *P* = 0.308). Two and three patients in the NBCA and PVA group, respectively, died due to recurrent haemoptysis. Other causes of death included cancer progression (n = 73), pneumonia (n = 20), and pulmonary embolism (n = 2). Forty-nine patients (27 vs. 22 in the NBCA vs. PVA groups; *P* = 0.284) were transferred to hospice care. The overall survival was not significantly different between the two groups (median survival, 226.0 vs. 113.0 days in the NBCA vs. PVA groups; *P* = 0.119) (Fig. 3b).

Table 3 demonstrates the results of the univariable and multivariable Cox proportional hazard analyses for identifying the predictors of HFS. On univariable analysis, the amount of haemoptysis, type of embolic material, and presence of coagulopathy before BAE were associated with HFS. Multivariable analysis including these factors showed that the use of PVA (hazard ratio, 3.452; 95% confidence intervals, 1.994–5.974; *P* < 0.001) and the presence of coagulopathy (hazard ratio, 2.916; 95% confidence intervals, 1.238–6.870; *P* = 0.014) were independent predictors of shortened HFS.

**Table 3:** Univariable and multivariable analyses of predictors for haemoptysis-free survival

Parameter	Univariable Cox analysis			Multivariable Cox analysis		
	HR	95% CI	*P*-value	aHR	95% CI	*P*-value^†^
Embolic material type						
PVA	5.095	2.646–9.812	< 0.001	3.452	1.994–5.974	0.001
Age (y)						
> 67	0.974	0.596–1.592	0.917			
Male sex	1.242	0.613–2.514	0.547			
Haemoptysis amount (mL)						
100–300	0.616	0.353–1.075	0.088			
≥ 300	0.941	0.465–1.906	0.866			
Hemodynamic instability						
Unstable	1.473	0.747–2.907	0.264			
Tumour size	1	0.991–1.009	0.996			
Cavitary lung lesion	1.189	0.563–2.513	0.650			
NSCLC	1.146	0.546–2.409	0.718			
Central tumour location	1.542	0.879–2.706	0.131			
Coagulopathy	2.470	1.053–5.796	0.038	2.916	1.238–6.870	0.014

aHR: adjusted hazard ratio; CI: confidence interval; HR: hazard ratio; NSCLC:s non-small cell lung cancer

^†^Determined using Cox analysis

## Discussion

As no studies have investigated the use of NBCA for BAE in patients with haemoptysis from primary lung cancer, this retrospective study compared the safety and efficacy of BAE performed using NBCA and PVA particles in patients with primary lung cancer. In this retrospective cohort, BAE using NBCA showed superior initial hemostasis (*P* = 0.002) with longer HFS (*P* < 0.001), shorter procedure time (*P* < 0.001), and reduced radiation dose (*P* < 0.001) than that using PVA particles. The use of PVA and the presence of coagulopathy were independent predictors of recurrent haemoptysis.

Compared with the clinical outcomes of BAE for benign diseases, those for lung malignancy are low (58.3%–89%); recurrence rates are also higher (20%–75%) [2, 5, 6, 10–12, 19]. Thus, BAE is considered a second-line option in lung cancer when bronchoscopy or conservative treatment is of limited efficacy [20]. Differences in the pathogenesis of haemoptysis may explain this discrepancy. In benign diseases, chronic inflammation or longstanding pulmonary ischaemia cause enlargement of the bronchial arteries [1, 7, 21]. Thus, cardiac output to the bronchial arteries can increase by up to 30% [22]. However, lung malignancy is characterised by fine neovascularisation in and adjacent to the neoplasm, tumour necrosis without a hypervascular environment, and early recruitment of feeders due to tumour angiogenesis [20, 23]. Tumour-feeding arteries are generally small, multiple, and have slow flow [4, 24]. Therefore, new embolic materials are required to overcome these limitations.

In this study, NBCA was superior to PVA in terms of immediate haemostasis (81.0% vs. 53.1%, *P* = 0.002). NBCA is semifluid, enabling advancement into small vessels to achieve complete occlusion [1, 3, 7]. The penetration of NBCA can be controlled according to the size and flow rate of target vessels by adjusting the mixing ratio of NBCA and lipiodol. PVA particles can easily aggregate, possibly resulting in incomplete embolisation proximal to the intended level [3]; appropriate delivery can be challenging owing to the unique target vessel characteristics in lung cancer that include multiple fine feeders with slow flow rates.

HFS was longer in the NBCA group than in the PVA group (median survival, 176.0 vs. 16.5 days; *P* < 0.001). Multivariable analysis also revealed that the use of PVA was an independent risk factor for haemoptysis recurrence (adjusted hazard ratio 3.452, confidence interval 1.994–5.974; *P* < 0.001). Most recurrent haemoptysis cases in the PVA group (81.8%) occurred within 1 month after BAE, suggesting that recanalisation is the primary mechanism of rebleeding in the PVA group [7]. Studies have consistently reported the occurrence of recanalisation after BAE using PVA [1, 7, 25, 26]. As intravascular polymerisation of NBCA inhibits recanalisation, the more durable embolic effect of NBCA may explain the superior long-term outcomes.

Coagulopathy is a well-known cause of haemoptysis [10, 27, 28]. Cancer-associated coagulopathy often occurs in lung cancer, and the consumption of platelets and clotting factors can induce life-threatening haemorrhage such as pulmonary bleeding [29]. In coagulopathic conditions, particulate embolisation is less likely to achieve complete haemostasis [30]. Studies on uterine artery embolisation for patients with coagulopathy showed superior durability of NBCA over PVA or gelatin sponge particles [31, 32]. These results may be attributed to the fact that polymerisation by NBCA is independent of the patients’ coagulation status [33]. Although not significant because of the small sample size, the hemostasis success rate in the NBCA group (four of six) was twice that of the PVA group (one of three) in coagulopathic patients.

The NBCA group had a 65% shorter procedure time and 75% less dose–area product than the PVA group. BAE procedures are time-consuming, as multiple feeding vessels must be selected and embolised completely to achieve a successful outcome. In addition, particulate embolisation requires continuous fluoroscopic monitoring of the injected particle/contrast mixture until complete embolisation is achieved. By contrast, the NBCA/lipiodol mixture is radio-opaque and quickly polymerises within a few seconds according to the mixing ratio. An operator can easily notice the exact extent of embolisation and determine the endpoint of the embolisation. No data regarding procedure time or radiation dose of BAE is available in the literature [2]; however, reducing the radiation dose to the patients and operators while maintaining clinical performance is mandatory [34]. Thus, the use of NBCA for BAE may have advantages over PVA in reducing procedure time and radiation exposure.

In this study, no grade 3–5 adverse events were observed in either group. There have been concerns that liquid embolic agents, such as NBCA, may increase the risk of non-target embolisation and tissue necrosis. A study performed a histological analysis of the lung lesion after BAE using NBCA [35]. The authors found that NBCA did not cause necrosis of the bronchial wall or lung parenchyma and only filled the lumen of the bronchial arteries. In this study, no patient experienced bronchial wall abnormalities or pulmonary infarction, which was confirmed on subsequent CT and/or bronchoscopy after BAE. However, the wedged position of the microcatheter tip hinders the antegrade flow of NBCA and may cause early backflow, possibly leading to unintended reflux of NBCA [3]. In addition, polymerised NBCA that adhered to the microcatheter tip could be a source of non-target embolisation [7]. Such complications were not observed in this study; however, care must be taken during the procedure using NBCA.

This study has several limitations. This was a single-centre retrospective study with inherent selection bias, and the embolic material was determined at the interventional radiologists’ discretion. However, the baseline characteristics, angiographic findings, and the number of embolised vessels were well balanced between the groups. Thus, randomised controlled trials are needed to validate the benefits of NBCA over conventional embolic agents. Moreover, the clinical outcomes of the PVA group were relatively poor (initial haemostasis rate: 53.1%) compared with those in previous studies (63%–77.5%) [5, 6, 12]. This may be explained by the finding that previous investigations included patients with malignancy and concomitant benign lung diseases. Many lung cancer patients may have benign diseases, causing haemoptysis and leading to an overestimation of BAE effects. Five patients with concomitant benign lung lesions in this study were excluded. Therefore, the actual results of BAE using PVA particles for haemoptysis in patients with primary lung cancer alone may be worse than those reported in previous studies. Lastly, although this study possibly had the largest cohort of this study type, the sample size was small.

### Interpretation

BAE using NBCA in patients with primary lung cancer showed superior initial haemostasis with longer HFS, shorter procedure times, and reduced radiation dose than PVA particles. No significant complications were observed in both groups, and the use of PVA and the presence of coagulopathy were independent risk factors of shortened HFS.

## Data Availability

The datasets used for the current study are available from the corresponding author on reasonable request.

## Abbreviations

BAE: Bronchial artery embolisation
CT: Computed tomography
HFS: Haemoptysis-free survival
NBCA: *n*-Butyl-2-cyanoacrylate
PVA: Polyvinyl alcohol
